# Case Report: Definitive radiotherapy for intrathyroidal thymic carcinoma with neuroendocrine differentiation

**DOI:** 10.3389/fonc.2026.1944330

**Published:** 2026-09-30

**Authors:** Jia Du, Jiankai Wang, Jianbing Song, Yunzhi Xu, Xiaoyu Duan, Hongyi Cai

**Affiliations:** 1 The First Clinical Medicine College, Gansu University of Chinese Medicine, Lanzhou, China; 2 Department of Radiotherapy, Gansu Provincial Hospital, Lanzhou, China

**Keywords:** definitive radiotherapy, immunohistochemistry, individualized treatment, intrathyroidal thymic carcinoma, neuroendocrine differentiation

## Abstract

Intrathyroidal thymic carcinoma (ITTC) with neuroendocrine differentiation is rare, and evidence for definitive radiotherapy in nonsurgical patients remains limited. A 70-year-old woman with a 1-year history of a neck mass and hoarseness was diagnosed with ITTC with neuroendocrine differentiation based on histopathological, immunohistochemical, and imaging findings. After declining surgery and systemic chemotherapy, she received definitive radiotherapy: 60 Gy in 30 fractions to the planning target volume, followed by a sequential boost of 6 Gy in 3 fractions to the gross primary tumor and involved lymph nodes, for a cumulative dose of 66 Gy in 33 fractions. No grade ≥3 acute treatment-related toxicities occurred. At approximately 2 months after radiotherapy, the sum of target-lesion diameters decreased from 54 mm to 42 mm, a 22.2% reduction. With no new lesions or unequivocal progression of non-target disease, the overall response was classified as stable disease according to RECIST 1.1. This case documents early radiographic tumor reduction following definitive radiotherapy for ITTC, although longer follow-up is required to assess the durability of disease control and late toxicity.

## 1 Introduction

Intrathyroidal thymic carcinoma, formerly known as carcinoma showing thymus-like differentiation (CASTLE), is a rare primary malignant epithelial tumor of the thyroid, accounting for less than 0.15% of all thyroid malignancies (1).Preoperative diagnosis can be challenging because of its insidious onset and nonspecific clinical and imaging features, particularly when the primary site and tumor origin are uncertain. More broadly, the preoperative evaluation of thyroid nodules remains challenging, as fine-needle aspiration biopsy may yield indeterminate or inconclusive cytological results, while routinely available clinical and laboratory parameters have limited diagnostic value (2).

Surgical resection remains the mainstay of treatment. However, evidence regarding the efficacy, radiation dose, and target-volume design of definitive radiotherapy for patients who do not undergo surgery remains limited, and no consensus has been established (3). The present case is distinctive because of its neuroendocrine differentiation and the use of definitive radiotherapy after the patient declined surgery. We describe its diagnostic features and clinical course to provide additional experience with the nonsurgical management of ITTC.

## 2 Case presentation

On September 3, 2025, a 70-year-old woman presented with a 1-year history of a neck mass accompanied by neck discomfort and hoarseness. Physical examination revealed a hard 4 × 3 cm mass in the lower neck. Her medical history included hypertension, coronary heart disease, and diabetes mellitus. Indirect laryngoscopy showed left vocal cord paralysis. Thyroid ultrasonography demonstrated a 46 × 23 mm hypoechoic nodule with relatively well-defined margins, classified as TI-RADS category 4 (**Figure 1**). Contrast-enhanced cervical CT showed thyroid enlargement with an enhancing mass causing focal tracheal compression, together with enlarged right cervical level IV and mediastinal lymph nodes. Laboratory testing showed mildly elevated total thyroxine (TT4), whereas thyroid-stimulating hormone (TSH) and thyroglobulin levels were within the reference ranges.

**Figure 1:**
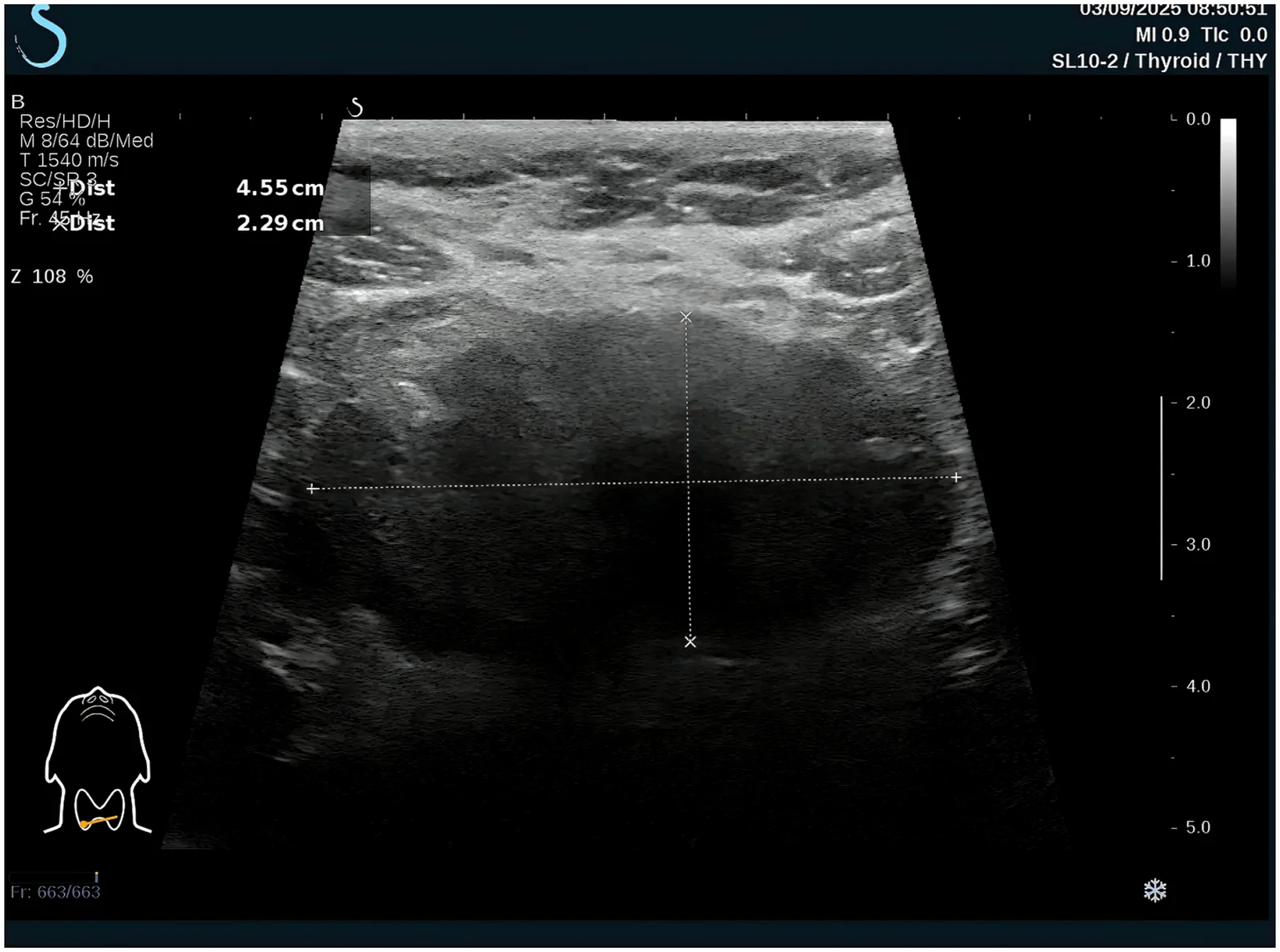
Thyroid ultrasonographic findings. Thyroid ultrasonography shows a 46 × 23 mm hypoechoic lesion within the thyroid gland.

Ultrasound-guided core needle biopsy of the thyroid mass showed infiltrative nests and cords of atypical cells with poorly differentiated squamous carcinoma-like morphology. Immunohistochemistry demonstrated positivity for p63, p40, CD5, CD117, CK-pan, and focal CK7, with negative staining for Pax-8, TTF-1, TPO, calcitonin, and PTH (**Figure 2**). Synaptophysin (Syn) and INSM1 were positive, whereas CD56 was negative; the Ki-67 proliferation index was approximately 20%. The morphology and immunophenotype supported thymic epithelial differentiation with neuroendocrine differentiation.

**Figure 2:**
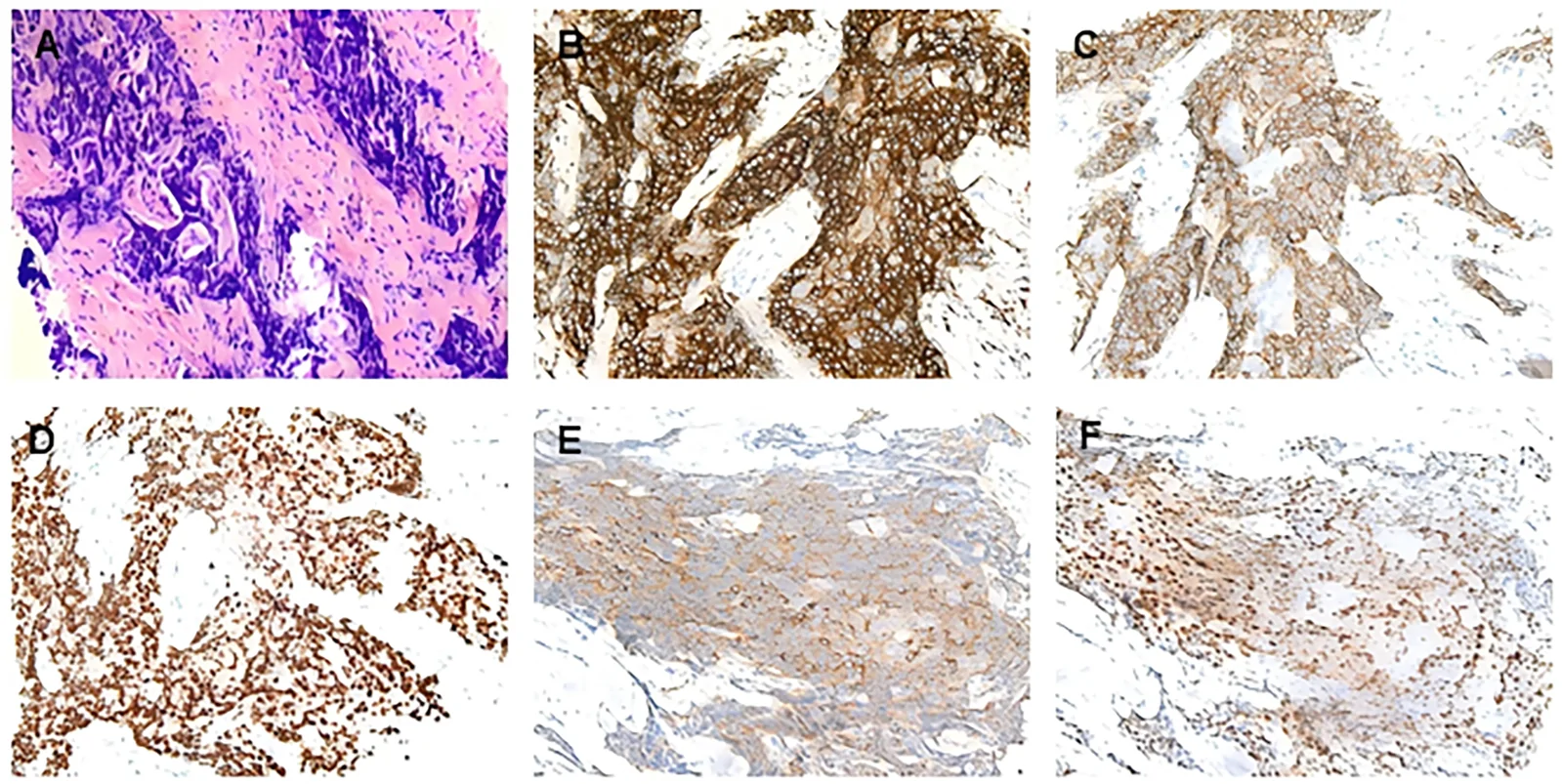
Histopathological and immunohistochemical findings of the tumor. **(A)** Hematoxylin and eosin staining showing poorly differentiated squamous carcinoma-like morphology. **(B–F)** Immunohistochemical staining for CD5, CD117, p63, synaptophysin (Syn), and INSM1, respectively; original magnification ×20.

On September 16, 2025, the patient was transferred to the Department of Radiotherapy. Re-evaluation of imaging confirmed upper-tracheal encasement with luminal stenosis and enlarged lymph nodes in the right supraclavicular region and mediastinal station 2R. Contrast-enhanced chest and abdominal CT showed no distant metastases or identifiable primary thymic tumor. Considering the intrathyroidal location, histopathological findings, and immunophenotype, a final diagnosis of intrathyroidal thymic carcinoma with neuroendocrine differentiation was established.

The patient declined surgery and systemic chemotherapy and therefore underwent definitive image-guided intensity-modulated radiotherapy on a Halcyon platform. Image guidance was used for treatment setup and positional verification. Radiotherapy was delivered in two sequential phases. In the first phase, the planning target volume (PTV) received 60 Gy in 30 fractions (2 Gy per fraction), with 95% of the prescribed dose intended to cover the target volume. This was followed by a sequential boost of 6 Gy in 3 fractions to the gross primary tumor (GTV) and radiologically involved lymph nodes (GTVnd), resulting in a cumulative prescribed dose of 66 Gy in 33 fractions to the gross disease. Radiotherapy was delivered from September 22 to November 6, 2025, over 46 calendar days.

Given the absence of established target-volume delineation guidelines for definitive radiotherapy of intrathyroidal thymic carcinoma, an individualized involved-site irradiation strategy was adopted. The GTV encompassed the radiologically visible primary thyroid tumor, whereas the GTVnd included the involved lymph nodes in the right supraclavicular region and mediastinal station 2R. A 5-mm margin was added around the GTV and GTVnd to account for potential microscopic extension and define a limited clinical target volume (CTV), followed by a further 5-mm expansion to generate the PTV (**Figure 3**). No elective nodal irradiation was delivered to radiologically uninvolved cervical or mediastinal nodal regions.

**Figure 3:**
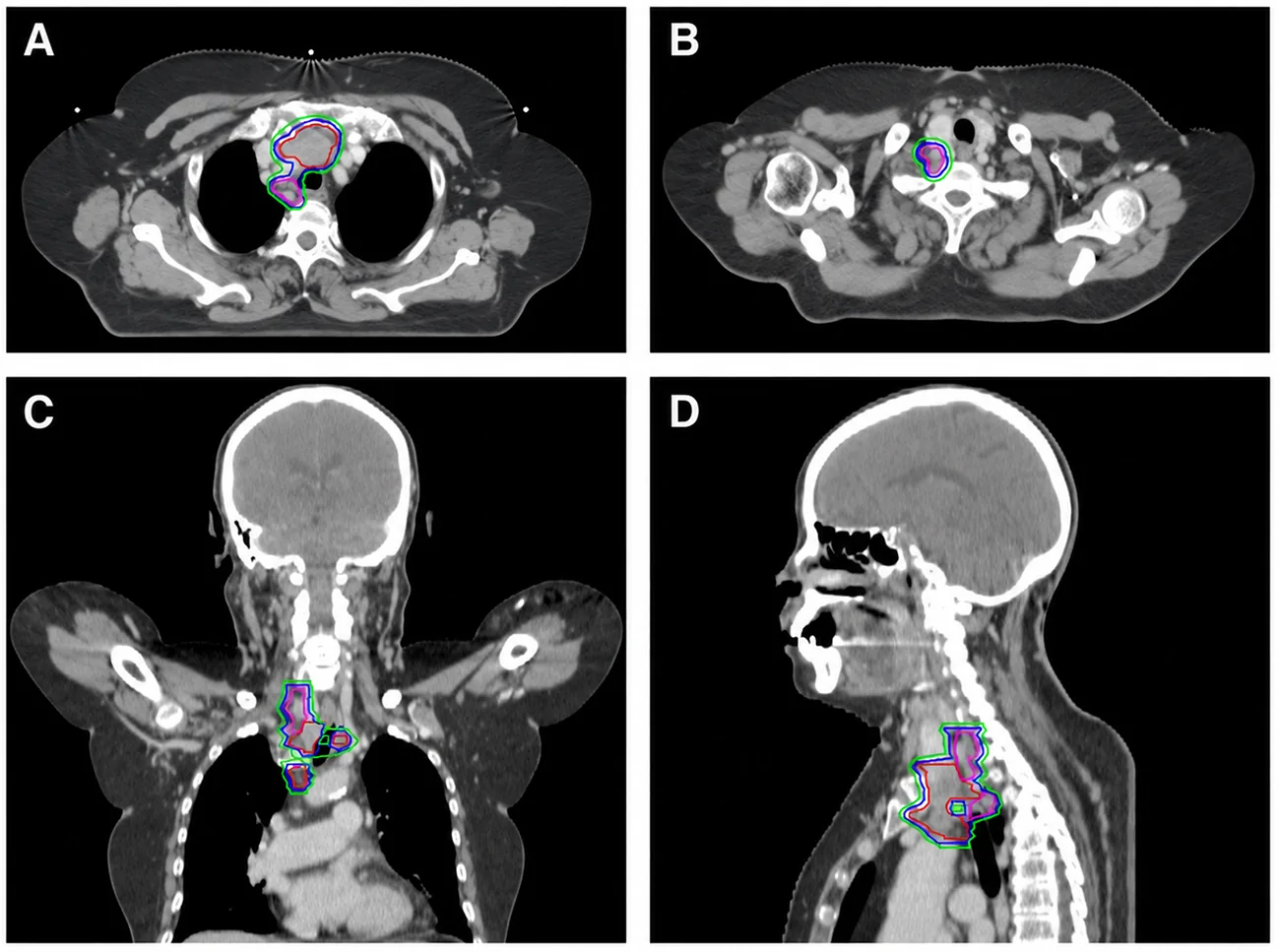
**(A, B)** Representative axial planning CT images at different anatomical levels. **(C)** Coronal planning CT image. **(D)** Sagittal planning CT image. The gross primary tumor (GTV, red), involved nodal disease (GTVnd, purple), clinical target volume (CTV, blue), and planning target volume (PTV, green) are shown.

Organs at risk included the spinal cord, bilateral lungs, heart, and esophagus. The recorded planning constraints were a maximum spinal cord dose of 40 Gy; whole-lung V20 <25% and V5 <50–55%; heart V30 <40% and V40 <30%; and esophageal V50 <50%. The cumulative dose–volume histogram and corresponding cumulative dose statistics are provided in **Supplementary Figure S1** and **S2**, respectively.

During radiotherapy, the patient experienced intermittent wheezing and received symptomatic inhalation therapy, with no documented acute airway obstruction, emergency airway intervention, or treatment interruption. The etiology of the wheezing could not be determined retrospectively. Acute toxicities were assessed according to CTCAE version 5.0 and included mild radiation-induced pharyngitis and grade 1 radiation dermatitis, both of which improved with symptomatic treatment.

Follow-up CT on January 12, 2026, showed continued reduction of the target lesions (**Figure 4**). According to RECIST 1.1, the primary thyroid lesion decreased from 39 to 31 mm in longest diameter and the mediastinal target lymph node from 15 to 11 mm in short-axis diameter. The sum of target-lesion diameters decreased from 54 to 42 mm (a 22.2% reduction). In the absence of new lesions or unequivocal progression of non-target disease, the overall response was classified as stable disease (SD). The patient reported improvement in hoarseness, although repeat laryngoscopy was not performed. Follow-up thyroid function showed normal TSH and T3, with persistently mildly elevated total T4 (174.11 nmol/L).

**Figure 4:**
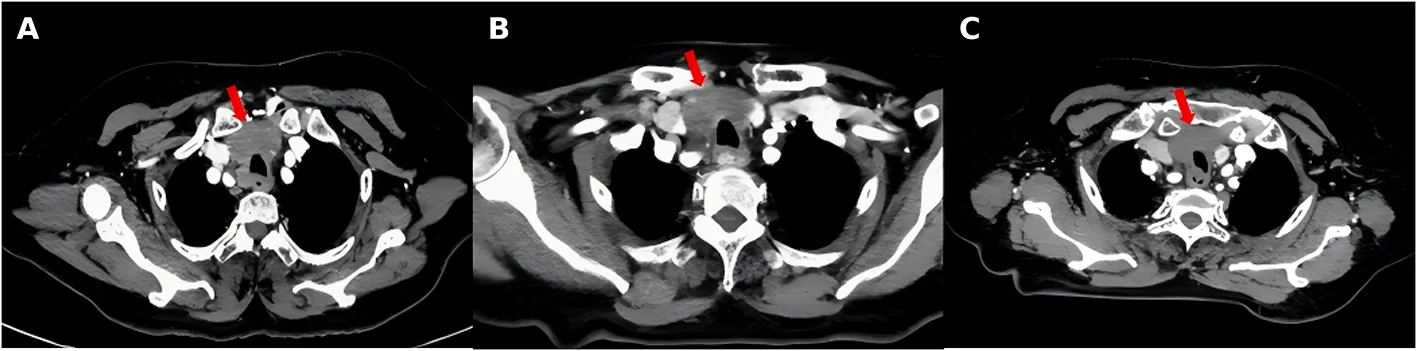
Radiological changes before and after definitive radiotherapy. **(A)** Pretreatment CT showing a soft-tissue mass involving the right inferior thyroid and retrosternal region with extension into the upper mediastinum. **(B)** CT obtained at the completion of radiotherapy showing tumor reduction compared with pretreatment imaging. **(C)** At the approximately 2-month assessment, the sum of target-lesion diameters had decreased by 22.2%, with an overall response of stable disease according to RECIST 1.1.

## 3 Discussion

ITTC is a rare malignant epithelial neoplasm arising within the thyroid gland and showing thymic differentiation. Its nomenclature emphasizes both the primary anatomical site and thymic lineage (1). ITTC is commonly located in the lower pole of the thyroid gland and usually presents as a unilateral solitary lesion (4, 5). Although ITTC generally exhibits relatively indolent biological behavior and a favorable prognosis (6), it demonstrates strong local invasiveness and a relatively high incidence of regional lymph node metastasis (7, 8). Because of its rarity and the lack of specific clinical manifestations and imaging features, diagnosis remains challenging.

Clinically, most patients present with a painless neck mass. When the tumor invades adjacent structures, symptoms such as dyspnea, dysphagia and hoarseness may occur (9). Thyroid function remains normal in the majority of cases. On ultrasonography, lesions are mostly hypoechoic or heterogeneously hypoechoic nodules, generally classified as TI-RADS category 4 or above. CT scans reveal soft-tissue masses with varying degrees of enhancement. On MRI, the lesions show isointense or hypointense signals on T1-weighted images and moderately hyperintense to hyperintense signals on T2-weighted images. The patient in this case initially presented with a neck mass and hoarseness, and imaging revealed a thyroid lesion with regional lymphadenopathy. However, a definitive diagnosis could not be established based on imaging findings alone and required pathological confirmation.

Review of the available literature indicates that the cytological features of ITTC may provide diagnostic clues, but cytology alone rarely permits a definitive diagnosis (10). Histologically, ITTC may show squamous carcinoma-like or basaloid morphology, occasionally with neuroendocrine differentiation. Immunohistochemistry is critical for determining tumor origin. ITTC typically expresses thymic epithelial markers, including p63, p40, CD5, and CD117, whereas thyroid-lineage markers such as Pax-8, TTF-1, and TPO are generally negative (11–15). In the present case, the diagnosis was based on an ultrasound-guided core needle biopsy rather than a surgical resection specimen; therefore, comprehensive histopathological assessment of the entire tumor was not possible.

During the diagnostic work-up, the biopsy showed poorly differentiated squamous carcinoma-like morphology, raising consideration of poorly differentiated thyroid carcinoma, anaplastic thyroid carcinoma, and primary squamous cell carcinoma of the thyroid. However, the combined immunophenotype, including CD5 and CD117 positivity and negativity for thyroid-lineage markers, favored thymic epithelial differentiation over a primary thyroid malignancy. Medullary thyroid carcinoma and parathyroid-derived tumors were less likely because calcitonin and PTH staining were negative. Although Syn and INSM1 were positive, the overall immunophenotype was not entirely consistent with a pure neuroendocrine carcinoma. Distinction from primary mediastinal thymic carcinoma with secondary thyroid involvement is discussed below.

It should be noted that ITTC closely resembles primary thymic squamous cell carcinoma in histological morphology and immunophenotype. Both tumors may show poorly differentiated squamous cell carcinoma-like features and express thymic epithelial markers, making differentiation by immunohistochemistry alone difficult (13). The key distinguishing factor is the presumed site of origin. In the present case, the tumor was predominantly located in the lower pole of the thyroid gland, and contrast-enhanced chest CT revealed no identifiable primary anterior mediastinal thymic tumor. Together with the pathological findings and immunophenotype, these imaging features supported an intrathyroidal origin and the diagnosis of ITTC. However, because PET-CT was not performed, an occult extra-thyroidal primary tumor could not be excluded with absolute certainty.

In addition, some ITTC cases express neuroendocrine markers such as Syn and CgA, and ITTC with neuroendocrine differentiation is relatively uncommon (16). In this case, immunohistochemical staining was positive for Syn and INSM1. CgA was not assessed in this patient. INSM1 is a recently recognized nuclear marker associated with neuroendocrine differentiation. It has high sensitivity and specificity for neuroendocrine tumors of the head, neck and other sites, serving as an important auxiliary indicator for identifying neuroendocrine differentiation (13, 17). Accordingly, the interpretation of neuroendocrine differentiation in this case was based on the available Syn and INSM1 positivity rather than a complete neuroendocrine immunohistochemical panel.

Regarding treatment, surgery remains the preferred therapeutic approach for ITTC (18). Radiotherapy also plays an important role in multimodality treatment, including postoperative adjuvant radiotherapy for patients with positive margins, residual disease, or a high risk of recurrence (18), definitive radiotherapy for locally advanced or unresectable disease (19), and palliative radiotherapy for symptom control (20). Previous case reports have described durable disease control and even complete remission after definitive radiotherapy or chemoradiotherapy in selected patients with locally advanced or unresectable ITTC (21, 22). However, the available evidence is limited to small retrospective series and individual case reports, and the optimal definitive radiation dose remains undefined. In the absence of disease-specific recommendations, the dose used in this case was selected with reference to dose ranges commonly used for thymic carcinoma, and a cumulative dose of 66 Gy in 33 fractions was delivered to the gross disease. Because the patient declined both surgery and systemic chemotherapy, radiotherapy was administered as the sole definitive local treatment.

With respect to target-volume design, no standardized approach has been established for ITTC. Previous reports have used different strategies, ranging from selective prophylactic irradiation, in which the central compartment was included while uninvolved lateral cervical regions were omitted (23), to extended-field irradiation covering bilateral cervical levels II–VI and upper mediastinal lymph nodes in locally advanced or unresectable disease (22). These heterogeneous approaches support individualized target-volume delineation based on the documented extent of disease and anticipated normal-tissue exposure.

In the present case, an individualized involved-site irradiation strategy was adopted. Treatment was limited to the radiologically evident primary tumor and involved lymph nodes, with a limited margin for potential microscopic extension, while radiologically uninvolved cervical and mediastinal nodal regions were not electively irradiated. Previous analyses have suggested that distant metastasis may represent an important pattern of failure in ITTC, whereas isolated regional nodal recurrence appears less frequent (23). Accordingly, and in the absence of additional radiologically involved nodes, elective nodal irradiation beyond the involved sites was not performed. At approximately 2 months after radiotherapy, the sum of target-lesion diameters decreased by 22.2%; however, the overall response remained stable disease according to RECIST 1.1, and no grade ≥3 acute treatment-related toxicity was observed. Given the short follow-up and single-patient nature of this report, these findings should be interpreted cautiously and should not be considered evidence of the long-term efficacy or generalizability of this target-volume strategy.

## 4 Conclusion

For selected patients with ITTC who are managed without surgery, definitive radiotherapy may be considered as a potential individualized treatment option. In this case, definitive radiotherapy was associated with short-term disease stability and manageable acute toxicity; however, longer follow-up and additional clinical evidence are required to assess long-term efficacy and safety.

## 5 Limitations

First, this single-patient report with limited follow-up cannot establish durable efficacy or late toxicity. Second, the individualized radiotherapy plan precludes conclusions regarding the optimal dose or target-volume design for ITTC. Third, diagnosis was based on core needle biopsy without PET-CT or CgA assessment, limiting comprehensive pathological and imaging evaluation.

## Data Availability

The original contributions presented in the study are included in the article/**Supplementary Material**. Further inquiries can be directed to the corresponding authors.

## References

[1] Rindi G Mete O Uccella S Basturk O La Rosa S Brosens LAA . Overview of the 2022 WHO classification of neuroendocrine neoplasms. Endocr Pathol. (2022) 33:115–54. doi: 10.1007/s12022-022-09708-2 35294740

[2] Aydoğdu YF Büyükkasap Ç Göbüt H Akın M . ABO blood group and Rhesus factor as potential markers in papillary thyroid cancer: a retrospective comparative analysis (2015-2023). Langenbecks Arch Surg. (2025) 411:25. doi: 10.1007/s00423-025-03920-4 41313453 PMC12662903

[3] Wang B Wang SS Huang WY Lin X He SF Zhao WX . Diagnosis and treatment strategies for intrathyroidal thymic carcinoma [in Chinese. Chin J Pract Surg. (2025) 45:1066–70. doi: 10.19538/j.cjps.issn1005-2208.2025.09.20

[4] Jiang L Zheng WH Chen C . Genomic variation associated with carcinoma showing thymus-like elements (CASTLE) in thyroid gland. Laryngoscope Investig Otolaryngol. (2022) 7:894–900. doi: 10.1002/lio2.805 35734076 PMC9194970

[5] Stanciu M Ristea RP Popescu M Vasile CM Popa FL . Thyroid carcinoma showing thymus-like differentiation (CASTLE): a case report. Life (Basel). (2022) 12:1314. doi: 10.3390/life12091314 36143350 PMC9503371

[6] Gurizzan C Zamparini M Volante M Tovazzi V Amoroso V Consoli F . Outcome of patients with intrathyroidal thymic carcinoma: a pooled analysis. Endocr Relat Cancer. (2021) 28:593–604. doi: 10.1530/erc-21-0123 34105516

[7] Anila KR Nair IS Nair LM Thomas S . Intrathyroidal thymic carcinoma not so indolent a neoplasm? Indian J Pathol Microbiol. (2024) 67:628–30. doi: 10.4103/ijpm.ijpm_232_23 38391347

[8] Ito Y Miyauchi A Hirokawa M Kihara M Onoda N Miya A . Clinicopathological features and outcomes of intrathyroidal thymic carcinoma: a single institution study. Endocr J. (2022) 69:1351–6. doi: 10.1507/endocrj.EJ22-0141 35768280

[9] Dang NV Son LX Hong NTT Nhung NTT Tung NT Quang LV . Recurrence of carcinoma showing thymus-like differentiation (CASTLE) involving the thyroid gland. Thyroid Res. (2021) 14:20. doi: 10.1186/s13044-021-00111-3 34399813 PMC8365953

[10] Okubo Y Sakai M Yamazaki H Sugawara Y Samejima J Yoshioka E . Histopathological study of carcinoma showing thymus-like differentiation (CASTLE). Malays J Pathol. (2020) 42:259–65. 32860379

[11] Wang X Zhang H Wang B Zhang L . Navigating the rare: a case report of intrathyroidal thymic carcinoma. Gland Surg. (2025) 14:570–6. doi: 10.21037/gs-2024-540 40256475 PMC12004328

[12] Weissferdt A Moran CA . Thymic carcinoma, part 1: a clinicopathologic and immunohistochemical study of 65 cases. Am J Clin Pathol. (2012) 138:103–14. doi: 10.1309/AJCP88FZTWANLRCB 22706865

[13] Gao Y Yang B . Intrathyroid thymic carcinoma: a comprehensive review of clinicopathological features and therapeutic strategies. Histol Histopathol. (2026) 15:25090. doi: 10.14670/hh-25-090 42136544

[14] Liu Z Teng XY Sun DX Xu WX Sun SL . Clinical analysis of thyroid carcinoma showing thymus-like differentiation: report of 8 cases. Int Surg. (2013) 98:95–100. doi: 10.9738/intsurg-d-12-00034.1 23701142 PMC3723174

[15] Lominska C Estes CF Neupane PC Shnayder Y TenNapel MJ O'Neil MF . CASTLE thyroid tumor: a case report and literature review. Front Oncol. (2017) 7:207. doi: 10.3389/fonc.2017.00207 28955658 PMC5602351

[16] Ren WH Dong K Huang XZ Zhu YL . Intrathyroidal thymic carcinoma exhibiting neuroendocrine differentiation: case report with cytomorphology, immunocytochemistry, and review of the literature focusing on cytology. Diagn Cytopathol. (2019) 47:1197–202. doi: 10.1002/dc.24281 31318494

[17] Mahalakshmi B Baskaran R Shanmugavadivu M Nguyen NT Velmurugan BK . Insulinoma-associated protein 1 (INSM1): a potential biomarker and therapeutic target for neuroendocrine tumors. Cell Oncol (Dordr). (2020) 43:367–76. doi: 10.1007/s13402-020-00505-9 32219703 PMC12990676

[18] Feng GC Liu Z Li HQ Zuo DH Sun HL Qiao LX . Clinical diagnosis and treatment analysis of 21 cases of intrathyroid thymic carcinoma. Zhonghua Yi Xue Za Zhi. (2024) 104:440–4. doi: 10.3760/cma.j.cn112137-20231008-00676 38326056

[19] Tsutsui H Hoshi M Kubota M Suzuki A Nakamura N Usuda J . Management of thyroid carcinoma showing thymus-like differentiation (CASTLE) invading the trachea. Surg Today. (2013) 43:1261–8. doi: 10.1007/s00595-013-0560-2 23543082

[20] Roka S Kornek G Schüller J Ortmann E Feichtinger J Armbruster C . Carcinoma showing thymic-like elements--a rare malignancy of the thyroid gland. Br J Surg. (2004) 91:142–5. doi: 10.1002/bjs.4510 14760659

[21] Huang X Li B Xu M Gong Y Feng H Wang X . Chemoradiotherapy as a pivotal therapeutic modality for inoperable intrathymic thyroid carcinoma: a case report and literature review. Med (Baltimore). (2025) 104:e44836. doi: 10.1097/MD.0000000000044836 41054169 PMC12499820

[22] Kovářová P Vojtíšek R Krčma M Daum O Baxa J Sukovská E . Inoperable CASTLE of the thyroid gland treated with radical radiotherapy with complete remission. Strahlenther Onkol. (2021) 197:847–53. doi: 10.1007/s00066-021-01800-6 34160632

[23] Kong FF Pan GS Zhai RP Du CR He XY Shen CY . Failure pattern and suggestions for target volume delineation of carcinoma showing thymus-like differentiation treated with intensity-modulated radiotherapy. BMC Cancer. (2022) 22:1083. doi: 10.1186/s12885-022-10171-9 36271328 PMC9585782

